# Bridging a Gap in Coherence: The Coordination of Comprehension Processes When Viewing Visual Narratives

**DOI:** 10.3390/vision8030050

**Published:** 2024-08-30

**Authors:** Maverick E. Smith, John P. Hutson, Mi’Kayla Newell, Dimitri Wing-Paul, Kathryn S. McCarthy, Lester C. Loschky, Joseph P. Magliano

**Affiliations:** 1 Department of Psychological & Brain Sciences, Washington University in St. Louis, 1 Brookings Drive, St. Louis, MO 63130, USA; mavericks@wustl.edu; 2 Department of Psychological Sciences, Kansas State University, Manhattan, KS 66506, USA; jhutson@ets.org (J.P.H.); loschky@ksu.edu (L.C.L.); 3 Department of Learning Sciences, Georgia State University, Atlanta, GA 30302, USA; mnewell6@student.gsu.edu (M.N.); dlwingpaul@gmail.com (D.W.-P.); kmccarthy12@gsu.edu (K.S.M.)

**Keywords:** Scene Perception and Event Comprehension Theory, event segmentation, bridging inferences, explanations, think-alouds

## Abstract

Scene Perception and Event Comprehension Theory (SPECT) posits that understanding picture stories depends upon a coordination of two processes: (1) integrating new information into the current event model that is coherent with it (i.e., mapping) and (2) segmenting experiences into distinct event models (i.e., shifting). In two experiments, we investigated competing hypotheses regarding how viewers coordinate the mapping process of bridging inference generation and the shifting process of event segmentation by manipulating the presence/absence of Bridging Action pictures (i.e., creating coherence gaps) in wordless picture stories. The Computational Effort Hypothesis says that experiencing a coherence gap prompts event segmentation and the additional computational effort to generate bridging inferences. Thus, it predicted a positive relationship between event segmentation and explanations when Bridging Actions were absent. Alternatively, the Coherence Gap Resolution Hypothesis says that experiencing a coherence gap prompt generating a bridging inference to close the gap, which obviates segmentation. Thus, it predicted a negative relationship between event segmentation and the production of explanations. Replicating prior work, viewers were more likely to segment and generate explanations when Bridging Action pictures were absent than when they were present. Crucially, the relationship between explanations and segmentation was negative when Bridging Action pictures were absent, consistent with the Coherence Gap Resolution Hypothesis. Unexpectedly, the relationship was positive when Bridging Actions were present. The results are consistent with SPECT’s assumption that mapping and shifting processes are coordinated, but how they are coordinated depends upon the experience of a coherence gap.

## 1. Introduction

**Figure 1:**
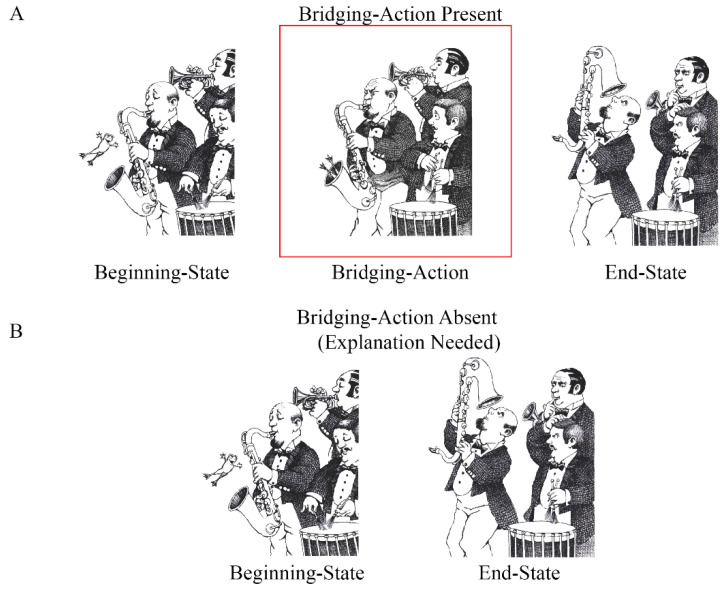
Illustration of the experimental manipulation. This target episode came from *Frog goes to dinner* [6], which was used in the present study. (**A**) shows the Bridging-Action present condition and (**B**) shows the Bridging-Action absent condition.

Visual narratives, such as wordless picture stories, convey events that make up a plot. However, some actions that make up the plot are not always explicitly depicted in the narrative [1]. To successfully comprehend a wordless picture story (we will hereafter refer to wordless picture stories as “picture stories”), viewers must respond to the missing information by generating inferences to connect pictures [2,3,4,5]. Consider the episode from *Frog goes to dinner* [6] shown in Figure 1A. The Beginning State picture shows a frog jumping in the air while a band plays their instruments. The highlighted Bridging Action shows the frog entering the saxophone and the End State shows the musician searching for the frog in the saxophone. Removing the Bridging Action, as shown in Figure 1B, creates a coherence gap, which likely disrupts viewers’ comprehension of the story (i.e., the saxophone player changes from playing to searching). To maintain coherence and interpret the End State as a continuation of the Beginning State in Figure 1B, viewers must infer the absent action from the content depicted in the End State (i.e., the saxophone player is searching for the frog because the frog jumped into the saxophone). Consistent with this example, researchers have demonstrated that readers maintain coherence by generating explanatory bridging inferences to account for why actions occur when they experience coherence gaps in their understanding [7,8,9,10,11].

Identifying when inferences are generated to comprehend narratives has been a central issue in research on narrative comprehension [7,12]. The End State picture in Figure 1B creates a coherence gap because there is no prior story event that explains why the saxophone player is looking in his saxophone. Such breaks in causal coherence provide a signal that an explanation is needed to bridge current and prior events [7]. Scene Perception and Event Comprehension Theory (SPECT) [13] provides mechanisms for the cognitive processes involved in recognizing and resolving coherence gaps. 

### 1.1. Scene Perception and Event Comprehension Theory

SPECT draws upon theories of narrative comprehension [7,14,15] and event cognition [16,17,18]. SPECT distinguishes between *front-end* and *back-end* processes. Front-end processes are the moment-to-moment perceptual processes that support scene, object, and motion perception and attentional selection, which determines where observers look next. Back-end processes support the construction of event models (one’s online mental representation of what is happening in the here and now) in working memory. We use the term *event model* to be consistent with Event Segmentation Theory [19] and Scene Perception and Event Comprehension Theory [13]. Radvansky and Zacks (2014) contrast event models with situation models, which, they argue, are mental representations extracted from discourse. The event model represents the spatiotemporal framework for when and where characters perform actions, the characters themselves, their goals, and the causal relations between actions, which make up events [15].

SPECT assumes that visual narrative comprehension depends on a bidirectional relationship between *front-end* and *back-end* processes. Several studies have focused on understanding how the current event model in the back-end influences the front-end processes of information extraction and attentional selection [13]. However, no studies have tested how the back-end processes are coordinated to support comprehension. These assumptions stem from the Structure Building Framework [14,20], which specifies three processes of event model updating: laying the foundation, mapping, and shifting.

Laying the foundation of an event model involves representing the first information specified in a story event, such as the setting (e.g., a restaurant), and the characters (e.g., the frog and band members) involved at the beginning of the event. Prior work found that people spend more time viewing the first picture in a story or in a new event [21] and that characters and clauses mentioned first in sentences are more accessible in memory than characters and clauses mentioned later [14,22]. Thus, initial representations serve as the foundation onto which subsequent information is integrated. 

The mapping process establishes coherence across story elements [20,22], which is essential for constructing coherent event models. Events are evaluated based on their situational relatedness to prior events across various dimensions, including agents, goals, space, time, and causality [15]. For example, in Figure 1A, each successive picture supports mapping because the actions involve the same characters. They are related to pre-established goals (the frog escaped from the boy and is scaring people at a restaurant) and they occur in the same spatiotemporal setting (a restaurant). Importantly, the actions are also causally related (e.g., the saxophone player is searching for the frog because it jumped into the saxophone). When there is a gap in coherence, such as in Figure 1B, readers construct explanatory bridging inferences. Magliano et al., (2016) found that removing Bridging Action pictures led to longer viewing times for the End State pictures. An eye tracking study [4] replicated this effect and showed that people spend the additional viewing time searching for content to help explain the gap. In addition, Magliano et al., (2016) found that people were more likely to mention the Bridging Action (i.e., the frog jumped into the saxophone) when it was absent (e.g., Figure 1B) than when it was present (e.g., Figure 1A). They speculated that this was because the *inferred* actions were more activated in the viewers’ event model due to the added effort expended to generate inferences. As such, explanatory bridging inferences support mapping [22].

Shifting occurs when one event ends and a new event begins. SPECT assumes that the process of event segmentation informs shifting such that shifting occurs at event boundaries. Researchers have used the event segmentation task to measure when shifting occurs [23,24]. Participants perform this task by explicitly indicating when they perceive boundaries between events. Ample correlational work shows that people segment and shift to create a new event model when there are changes in motion [25,26,27], situational features (e.g., characters, space/time, causality) [28,29,30,31], or the narrative’s global structure [32]. 

Laying the foundation, mapping, and shifting are presumed to be iterative processes that occur as a story unfolds. SPECT, which extends the Structure Building Framework, assumes that these processes inform one another. For instance, shifting occurs when mapping is not possible; however, this assumption was never tested experimentally, as we outline in the next section. 

### 1.2. Back-End Processes at Coherence Gaps

According to SPECT, both the mapping process of bridging inference generation and the shifting process of event segmentation are engaged when viewers perceive coherence gaps. However, the interplay between these processes at coherence gaps is unclear. Prior work informs two competing hypotheses about how they may be associated. 

The first is a Computational Effort hypothesis. It predicts a positive association between explanations and segmentation when Bridging Actions are absent. Magliano et al., (1999) identified the extent to which people generate explanations when there are situational changes in time, space, causality, characters, and goals. Such changes often, but not always, trigger the perception of a new event (i.e., shifting) [28,29,30,31]. They found a positive relationship between the number of situational changes and the number of explanations [33]. Consequently, perceiving a coherence gap may lead a person to perceive an event boundary (i.e., shifting/segmenting), which, in turn, encourages one to engage in the additional computational effort to generate explanations and bridge the coherence gap at event boundaries. 

Similarly, Brich et al., (2024) examined changes in viewing time and event segmentation by manipulating the presence of Bridging Action pictures using the *A Boy, a Dog, a Frog* picture stories. They replaced half of the Bridging Action pictures, like those shown in Figure 1B, with a blank screen [34]. Following the blank, either the End State depicted actions taken from an unrelated moment in the picture story (necessitating segmenting/shifting processes) or the End State portrayed a continuation of the Beginning State (necessitating bridging inference/mapping processes). As expected, both the likelihood of segmenting and viewing time were higher when the End State depicted a new episode compared to a continuation of the Beginning State, which they interpreted as evidence of shifting. Importantly, they also found a modest increase in both the viewing time and event segmentation in the Bridging-Action *absent* condition compared to the *present* condition when the End State depicted a continuation of the Beginning State. They interpreted this modest increase as evidence of mapping and the computational effort needed to bridge between pictures. Their results suggest that mapping and shifting are distinct processes; however, they did not explicitly test how the two are related at coherence gaps. 

The alternative hypothesis is a *Coherence Gap Resolution hypothesis*. In contrast to the Computational Effort hypothesis, the Coherence Gap Resolution hypothesis predicts a *negative* association between event segmentation and explanations when Bridging Actions are absent. This hypothesis says that people attempt to generate a bridging inference when they experience a coherence gap to maintain a coherent event model. If they are successful in generating the bridging inference, the mapping process is successful, eliminating the need to create a new model (i.e., shifting/segmenting). Thus, according to this hypothesis, people will only perceive an event boundary at a coherence gap when they *fail* to generate an explanation for it (i.e., a failure of the mapping process). Thus, generating a bridging inference should reduce the likelihood of perceiving an event boundary (i.e., shifting/segmenting). 

Consistent with this hypothesis, Papenmeier et al., (2019) showed participants Beginning-State video clips, taken from soccer games, of a player running toward a ball before kicking it. Half of the participants saw the player contact the ball and half did not. Participants then saw an End-State clip that either depicted a causal continuation of the Beginning State (i.e., the ball flying) or a clip that depicted a non-causal continuation of the Beginning State (i.e., the audience). They found that participants were less likely to perceive a new event and they were more likely to falsely detect the moment of contact with the ball, which was not shown, when the End State depicted a causal continuation of the Beginning-State clip [35]. One explanation is that viewers generated a bridging inference (e.g., inferring the kick from the End-State clip) to map across the coherence gap, which reduced the likelihood of perceiving an event boundary and shifting to create a new event model. However, Papenmeier et al., (2019) did not explicitly test this hypothesis.

### 1.3. Current Experiments and Hypotheses

We used the *three-pronged method* [36], which coordinates concurrent constructed responses (i.e., verbal reports by participants), behavioral measures (e.g., segmentation judgments), and theories of comprehension to draw conclusions about the processes that occur during coherence breaks. We collected event segmentation data in Experiment 1 and responses to think-alouds in Experiment 2. Participants in both experiments viewed the same picture stories. Critically, we manipulated the presence/absence of Bridging Actions in target episodes within each story.

In Experiment 1, we used the event segmentation task [23] to assess shifting. Given that people segment when there are changes in situational continuities, such as causality [28,29,30,31], we predicted that participants would be more likely to segment on End State pictures when Bridging Action pictures were absent than when they were present [34]. However, prior work has also shown that the gaps used in this study can be resolved with bridging inferences [4,5,37]. Thus, it was also possible that the absence of Bridging Action pictures would not affect event segmentation.

In Experiment 2, we analyzed think-aloud responses for comprehension strategies using a coding scheme inspired by Trabasso and Magliano (1996). Given that a previous study found that people were more likely to mention the action depicted in the foreground of the Bridging Action picture when the Bridging Action picture was *absent* than when it was present [5], we predicted that there would be more explanations—statements that explain why actions occurred—when Bridging Action pictures were absent. In addition, the coding scheme enabled us to also explore how coherence gaps affected a broader range of strategies in a set of exploratory analyses.

More importantly, we examined the relationship between the frequency of explanations in Experiment 2 and the likelihood of segmentation in Experiment 1. We tested two competing hypotheses that enabled us to refine SPECT. The Computational Effort hypothesis says that explanations during mapping are indicative of the cognitive effort needed to bridge a coherence gap, which triggers shifting [33]. Thus, it predicted a positive relationship between the likelihood of segmenting and the production of explanations on End State pictures when Bridging Actions were absent. Alternatively, the Coherence Gap Resolution hypothesis says that explanations help resolve perceived coherence gaps, which eliminate the need to shift to create a new event model [7,8,9,10,22]. Thus, it predicted a negative relationship between the likelihood of segmenting and the production of explanations on End State pictures when Bridging Actions were absent.

We also explored the relationship between segmentation and the other strategies revealed in the coding scheme, such as predictions, associations, and paraphrases when they occurred at a high enough frequency to be quantitatively analyzed. We assumed they could inform SPECT; however, we did not have theoretically motivated hypotheses about their association with segmentation.

## 2. Experiment 1

### 2.1. Method

#### 2.1.1. Participants

Forty-eight students enrolled in introductory psychology courses (35 identified as female, *M_age_* = 19.11, *SD* = 3.53) at Kansas State University participated for course credit. We collected data from 3 additional participants, but we excluded their data from the analysis either because they segmented every picture in the event segmentation task (*N* = 2) or because of low visual acuity (20/30) as determined from the Freiburg Visual Acuity and Contrast Test (*N* = 1) [38]. All the participants were native English speakers and all signed an informed consent form prior to participating.

We ran a power analysis using the viewing time data from Hutson et al., (2018). We started by fitting a linear mixed model with the log of viewing time on the End State picture as the dependent variable. We treated the Bridging Action presence as a fixed effect and the participant, episode (described below), and the image nested within each episode as random effects. We also allowed the manipulation of the Bridging Action presence to vary for each participant as a random slope effect. We reduced the estimate for the Bridging Action presence effect by 2/3rds (*β* = −0.15 × 2/3 = −0.09) and then we ran a simulation-based power analysis using the mixedpower package in R [39]. At an alpha of 0.05, the power analysis suggested we needed a sample of 40 participants to detect a main effect of Bridging Action presence at 90% power. Thus, we should be adequately powered to detect an effect on viewing time.

#### 2.1.2. Materials

Participants viewed six picture stories (ranging from 24 to 26 images each) selected from picture story books written by Mercer Mayer [6,40,41,42,43,44]. We used the same pictures as Magliano et al., (2016). Magliano and colleagues edited the original pictures to reduce their complexity because the original pictures contained a considerable number of background details that were inconsistent across pictures and stories.

Participants advanced through each picture story one picture at a time. Unknown to participants, each of the six stories contained four target episodes (like those shown in Figure 1). We predicted that viewers would have to generate a bridging inference for the foregrounded actions to close the gap in each episode when the Bridging Action picture was absent. Each episode consisted of three-images, (1) a Beginning State, (2) a Bridging Action, and (3) an End State, for a total of 24 target episodes per subject. We counterbalanced the presence of the Bridging Action pictures as follows. We labeled the “Bridging-Action present” condition as “A” and the “Bridging-Action absent” condition as “B” for six possible orders: (1) BAAB, (2) ABBA, (3) ABAB, (4) BABA, (5) AABB, and (6) BBAA. We combined the six presentation orders with the six pictures stories in a 6 × 6 Latin square, which resulted in 36 combinations.

#### 2.1.3. Procedure

Using chin rests, participants viewed the pictures in the stories at a distance of 53 cm on Samsung SyncMaster 957 MBS monitors running at 85 Hz and 10,124 × 768 pixel resolution. Participants advanced through each of the picture stories by pressing a button on a computer keyboard. Participants could not return to previous pictures. Participants performed the event segmentation task on each story immediately after viewing it. We collected segmentation data after participants viewed the entire story, because we also collected viewing time on each picture, and we did not want the additional segmentation response to confound the viewing time measure. Further, prior work demonstrated that the segmentation task is reliable within and between viewers [21,45,46]. To segment the story, participants saw thumbnails of all the pictures on a single screen and they clicked on pictures they judged as indicating the starting of new events. We modified the instructions used by Magliano et al., (2012) [47]. The instructions were as follows:

*We want you to identify when you think that the situation has changed in the story. Click on the picture that you judge to be the start of a new situation. How you define a change in the situation is up to you. Please keep in mind that most stories contain multiple situations that change. As such, you should be making multiple judgments when viewing a story.*

We did not show participants an example of a change in the situation. A red box appeared around each selected picture. Participants wrote a brief 4–5 sentence summary of each story after viewing and segmenting it. We did not analyze these summaries because their purpose was to encourage participants to comprehend the picture stories, and the summaries were brief (*M*_Sentences_ = 6.50, *SD*_Sentences_ = 2.22).

### 2.2. Results

We conducted all analyses in R (Version 4.2.0). We ran all the mixed models using the ‘lmer’ and ‘glmer’ functions [48]. We checked model convergence using the ‘checkconvergence’ function [49]. We planned to remove data when pictures were viewed less than 200 milliseconds prior to running the analyses (the duration of the average fixation on these images) [4]; however, we did not remove any data because all the End State pictures were viewed for more than 200 milliseconds.

#### 2.2.1. Analysis of Event Segmentation

To evaluate how removing Bridging Action pictures affected event segmentation, we conducted a logistic mixed-effects model with the segmentation response (Segmented = 1, Did not segment = 0) on the End State picture as the outcome and the presence/absence of the Bridging Action as a fixed effect. We determined the random effect structure of the model by comparing three different models with different random effect structures using a likelihood-ratio test [50,51]. We started by fitting the maximal model [52] and then we reduced it by removing the random slope effects and then the random intercept effects until the more complex model was a statistically better fit of the data than the reduced model. We retained the reduced model when it did not significantly differ from the more complex model. See the Supplementary File for model comparisons.

**Figure 2:**
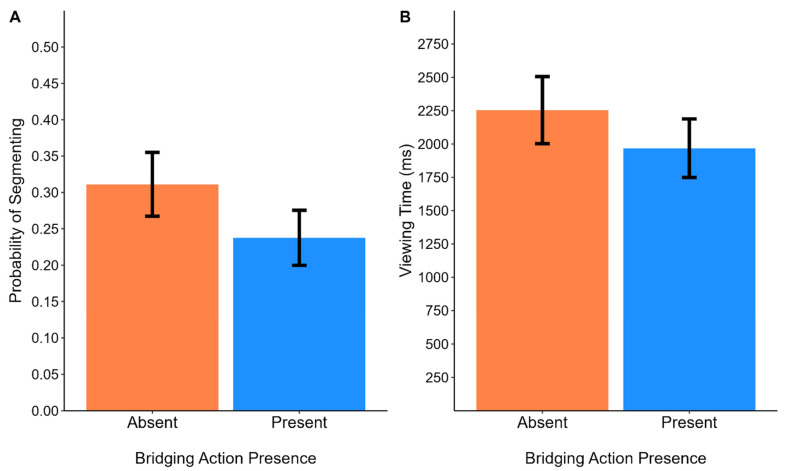
Probability of segmenting (**A**) and viewing times (**B**) on End State pictures. Bars show the means estimated from mixed-effects models. Error bars correspond to 1 ± standard error from the estimated means.

The model selection procedure led to the retention of the subject and the image nested within each episode as random intercept effects. In Wilkinson notation, this model is written as follows: *Segmentation* ~ 1 + *Bridging-Action Presence* + (1|*Participant*) + (1|*Episode:Image*). As shown in Figure 2A, participants were more likely to segment on End State pictures when the Bridging Action was absent (*M* = 0.31, *SE* = 0.08) than when it was present (*M* = 0.24, *SE* = 0.04): *β* = −0.37, *SE* = 0.14, *z* = −2.67, *p* = 0.008, odds ratio = 1.45, and 95% CI = [0.74–0.80]. To assess how well the logistic mixed model was able to classify whether an image was identified as an event boundary and to provide an estimate of the size of the effect, we calculated the area under the receiver operating characteristic curve using the model’s estimated values and the observed data [53]. The logistic model did well, AUC = 0.77. Additionally, we derived Bayes factors by comparing the AIC values from the alternative model, which contained the fixed effect of Bridging Action presence, to a null model, which did not [54]. We found strong evidence in favor of the alternative, BF = 12.71. Together, the results replicate that of Brich et al., (2024)

#### 2.2.2. Analysis of Viewing Time

Given the positive skew of viewing time, we modeled it with a Gamma regression [55]. We linearized the parameters in the model using a log link function. The model selection procedure led to the retention of the maximal model. This model is written as follows: *Viewing Time* ~ 1 + *Bridging Event Presence* + (1 + *Bridging-Action Presence*|*Participant*) + (1|*Episode*) + (1|*Episode:Image*). As shown in Figure 2B, participants viewed Bridging Action pictures longer when Bridging Action pictures were absent (*M* = 2254, *SE* = 252) than when they were present (*M* = 1968, *SE* = 219): *β* = −0.14, *SE* = 0.03, *t* = −4.07, *p* < 0.001, *d* = 0.25 and BF = 15.49. We calculated effect sizes for the generalized linear mixed-effects models using formulas provided by Brysbaert and Stevens (2018) [56]. We divided the absolute value of the coefficient for the main effect of Bridging Action presence by the square root of the sum of the variances for each random effect. Taken together with the analysis of segmentation and consistent with prior work [2,5,34,37], removing Bridging Actions affects viewers’ understanding of picture stories.

### 2.3. Discussion

We found that coherence gaps (i.e., removing the Bridging Action picture), which are presumed to be resolved readily with a bridging inference, are associated with an increased likelihood of segmenting and increases in processing effort in college students. This replicated work showing that experimentally manipulating the presence of pictures in picture stories affects shifting (i.e., segmentation responses) [34]. However, these results lead to an interesting question. Specifically, prior work has also shown evidence that students can bridge across these coherence gaps with an explanation (i.e., a mapping process) [5]. We ran Experiment 2 to explore how manipulating Bridging Action presence affects both mapping and shifting.

## 3. Experiment 2

College students thought aloud as they viewed wordless picture stories. Given that people are more likely to mention Bridging Actions on End State pictures [5] and to search them for inference-relevant content to explain missing Bridging Actions [4], we hypothesized that participants would generate more explanations on End State pictures when Bridging Actions were absent.

More importantly, we used the strategies participants used in Experiment 2 to predict segmentation behavior in Experiment 1. The Computational Effort Hypothesis predicts a positive relationship between explanations and segmentation. Alternatively, the Coherence Gap Resolution Hypothesis predicts a negative relationship between explanations and segmentation.

### 3.1. Method

#### 3.1.1. Participants

Twenty-one college students enrolled in courses in the Department of Learning Sciences (17 identified as female, *M_age_* = 27.72, *SD* = 10.88) at Georgia State University participated for course credit. We collected data from 4 additional participants; however, we excluded their data from the analysis because they either did not complete the experiment (*N* = 1) or were non-native English speakers (*N* = 3). Although the study did not involve text, prior work suggests differences in the content and quality of think-alouds produced by native and non-native speakers. The university Institutional Review Board approved all procedures, and all participants provided informed consent.

We ran a power analysis using the probability of mentioning the Bridging Action from Magliano et al., (2016). We fit a logistic mixed model with the likelihood of mentioning the Bridging Action (1 = mentioned Bridging Action, 0 = did not mention Bridging Action) on the End State picture as the dependent variable. We retained the maximal model. As in Experiment 1, we reduced the estimate for the Bridging Action presence effect by 2/3rds (*β* = 1.04 × 2/3 = 0.69) and then we ran a simulation-based power analysis [39,57] with 1000 simulations as in Experiment 1. At an alpha of 0.05, the power analysis suggested we needed a sample of 20 participants at 80% power to detect an effect of Bridging Action presence on think-aloud responses.

#### 3.1.2. Procedure

Participants viewed the same picture stories used in Experiment 1 as part of a picture book. Given practical limitations for data collection, participants viewed the stories from a comfortable distance from the book. Participants were free to move their heads as they wished, and we did not control the viewing distance as we did in Experiment 1. The experimental instructions were as follows:

*What you will do is view picture stories and you will “think out loud” as you view them. When you think aloud, you say the thoughts that come to mind after you view each picture in the stories. It is like turning up the volume on what you are thinking in your head as you make sense of the story in the pictures.*

Participants practiced thinking aloud with an unrelated picture story depicting a card game where one player gets caught cheating. The first 5 pictures of the practice included example think-aloud protocols demonstrating a variety of strategies (paraphrasing [picture descriptions and picture narrations], explaining, predicting, etc.). We constructed the examples so that we did not emphasize any strategies more than others (see OSF for the full instructions, practice story, and examples). Participants practiced thinking aloud for the remaining 6 pictures in the practice story. During the experimental stories, we told participants to think aloud after viewing each picture. They did not have the ability to return to previous pictures. Before turning each page in the picture book, a research assistant read the story title aloud and stated the upcoming picture number. We recorded and then transcribed each participant’s audio.

#### 3.1.3. Think-Aloud Coding

The think-aloud responses were coded using a rubric adapted from Trabasso and Magliano, (1996) and the process of coding reflected standard practice in the analysis of verbal protocols. Think-aloud responses were parsed into idea units (clauses containing a verb phrase) and each idea unit was classified into one of five strategies. These codes (explanation, prediction, association, picture description, picture narration, metacognitive statement or question, evaluation, an error) reflected common comprehension strategies that readers use to comprehend text and viewers use to make sense of picture stories. Table 1 shows descriptions of each strategy and sample clauses. We report these to illustrate the different strategies. Additional details about the coding scheme can be found in the Supplementary File. To examine our specific hypotheses, the analyses focus on two strategies: explanations and picture paraphrases. Explanations are statements about why actions occurred and picture paraphrases are statements about the objects, characters, their spatial arrangement in the pictures, and the actions they performed.

Four raters were trained on the rubric using a random subset of 5% of the data. When the coders determined that they understood the coding scheme, they then shifted to the reliability phase. In the reliability phase, 10% of the protocols were coded independently and Fleiss’ Kappa was computed. Discrepancies between raters were adjudicated through discussion. The team then independently coded another 10% of the data in the same manner. Approximately 69% of the participant responses were coded during the practice phases, and the raters achieved an acceptable level of reliability in the final phase of reliability (*Fleiss’ Kappa* = 0.78).

### 3.2. Results

The frequencies of the different strategies are shown in Table 2. We calculated the mean number of each strategy used by each participant when the Bridging Action was absent versus when it was present and then we calculated the mean frequencies across participants (we also analyzed the think-aloud responses using the proportion of explanations. The results were analogous to those that are reported here). Table 2 shows that we classified most idea units as picture paraphrases and explanations. We did not analyze the other strategies further because they did not occur frequently enough for us to analyze.

#### 3.2.1. Analysis of Think-Aloud Strategies

##### Analysis of Explanations

We modeled the frequency of explanations on the End State picture when the Bridging Action was present versus when it was absent using a Poisson mixed-effects model. To control for verbosity on a given End State, we included the total number of idea units each participant produced on the End State as a continuous covariate. We used a log link function to linearize the parameters in the model. There were more explanations when the Bridging Action was absent (*M* = 1.67, *SE* = 0.15) than when it was present (*M* = 1.42, *SE* = 0.13) (the means shown in Table 1 are raw means. The means in the text denote means from the estimated regression equation, after back-transforming the dependent variable on the log scale); *β* = −0.16, *SE* = 0.07, *z* = −2.34, *p* = 0.02, *d* = 0.43, and BF = 5.50.

##### Exploratory Analysis of Picture Paraphrasing

We also explored how the Bridging Action’s presence/absence influenced picture paraphrasing. We modeled the frequency of picture paraphrases the same way we modeled explanations. Even though missing Bridging Actions affected the number of explanations participants generated, we did not find evidence that missing pictures influenced scene paraphrasing after statistically controlling for verbosity; *β* = 0.09, *SE* = 0.05, *z* = 1.73, *p* = 0.08, and *d* = 0.38. The Bayes factor provided inconclusive evidence in favor of the alternative hypothesis; BF = 1.63.

#### 3.2.2. Analysis of Event Segmentation and Think-Aloud Strategies

The analyses of the think-aloud strategies in Experiment 2 and the analysis of event segmentation in Experiment 1 afforded testing hypotheses about the relationship between think-aloud strategies and event segmentation and, thus, the relationship between mapping and shifting.

##### Analysis of Event Segmentation and Explanations

**Figure 3:**
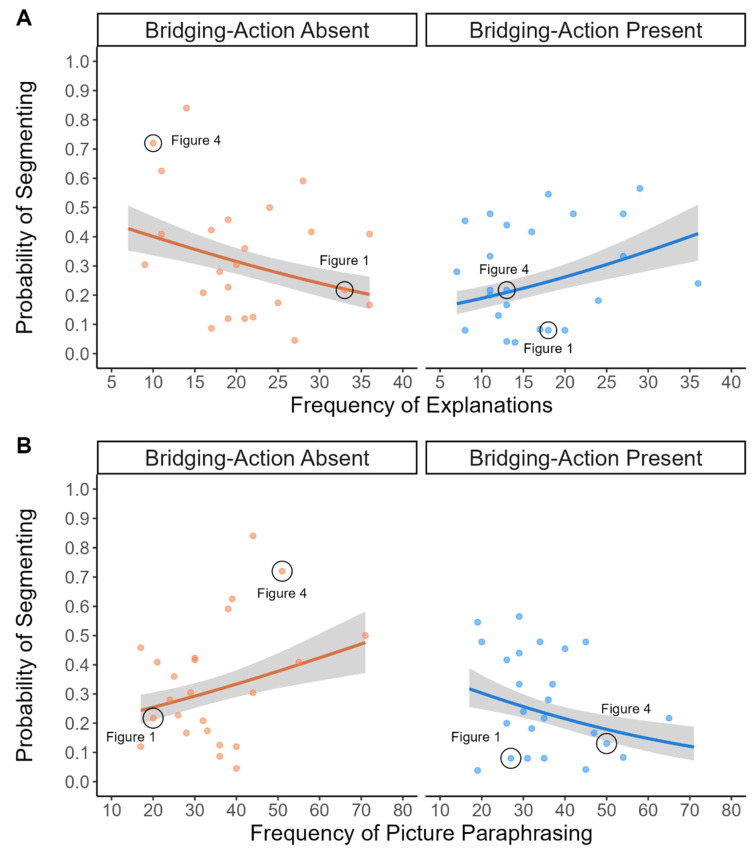
Probability of identifying the End State picture as an event boundary when the Bridging Action was absent or present as a function of the frequencies of (**A**) explanations and (**B**) picture paraphrasing. Dots represent the proportion of participants who identified each picture as the start of a new event. The black circles drawn around the two data points in each panel correspond to the target episodes shown in Figure 1 and Figure 4. The lines are estimates from mixed-effects models. Error ribbons correspond to 1 ± standard error.

Before running the analyses, we summed the frequency of explanations for each End State picture separately for when the Bridging Action was absent and when it was present for each picture. We then reran the logistic mixed-effects model of event segmentation reported in Experiment 1 using the fixed effects of Bridging Action presence (effect coded as: absent = −1 and present = +1), the frequency of explanations (grand mean centered to remove nonessential multicollinearity between predictors), and their interaction. As shown in Figure 3A, we found a significant interaction between the presence of the Bridging Action and the number of explanations on the End State: *β* = 0.04, *SE* = 0.01, *z* = 3.48, *p* < 0.001, odds ratio = 1.04, BF = 19.98, AUC = 0.77, and 95% CI = [0.75–0.80]. Consistent with the Coherence Resolution hypothesis, we found a negative association between explanations and segmentation when the Bridging Action was absent: *β* = −0.03, *SE* = 0.02, and 95% CI = [−0.07, −0.002]. Thus, viewers were less likely to segment target episodes that afforded more explanations. Unexpectedly, the relationship was positive when the Bridging Action was present: *β* = 0.04, *SE* = 0.02, and 95% CI = [0.006, 0.08]. This pattern was consistent with the Computational Effort hypothesis, albeit we predicted this pattern in the Bridging-Action absent condition.

##### Exploratory Analysis of Event Segmentation and Picture Paraphrasing

We also explored the relationship between picture paraphrasing and event segmentation. As shown in Figure 3B, we found a significant interaction between picture paraphrasing and the presence of the Bridging Action: *β* = −0.02, *SE* = 0.007, *z* = −2.95, *p* < 0.001, odds ratio = 1.02, BF = 3.68, AUC = 0.77, and 95% CI = [0.74–0.80]. Follow-up tests showed that the relationship between segmentation and picture paraphrasing was positive when the Bridging Action was absent—*β* = 0.01, *SE* = 0.02, and 95% CI = [−0.001, 0.04]—and negative when the Bridging Action was present—*β* = −0.02, *SE* = 0.01, and 95% CI = [−0.05, 0.004]. However, the simple slopes were not significantly different from zero.

### 3.3. Discussion

We started by inspecting the frequency of each strategy (Table 1). Participants primarily described the events conveyed in the pictures and explained how those events fit into the story. This finding was consistent with results from the discourse comprehension literature [7,11,33]. However, in the context of reading narrative texts, explanations occurred much more frequently than paraphrasing [11]. An explanation for such differences across media is unknown; however, they may have to do with differences in the affordances conveyed in picture stories versus narrative texts [58,59]. Specifically, events described in a text can be conveyed linguistically with a level of precision that may make deriving paraphrases less resource demanding. As such, more resources can be devoted to explanatory processes that support mapping story elements. In contrast, picture stories convey a complex set of actions in any given picture and viewers must extract those actions from the output of front-end perceptual processes; therefore, paraphrases dominated the ideas produced while viewing picture stories.

In addition, participants produced very few predictions. This was surprising, given theoretical proposals that emphasize the role of predictions in event comprehension such as Event Segmentation Theory [19]. Nevertheless, the finding that participants produced few predictions was consistent with prior work showing that predictions happen infrequently and tend to occur only when possible narrative outcomes are highly constrained [60]. The outcomes in the End State pictures may not have been constrained enough to provoke predictions. Alternatively, think-alouds may not have been sensitive to predictive inferences generated on End State pictures.

There were more explanations when the Bridging Action was absent than when it was present. This finding is consistent with theories of comprehension that emphasize explanatory inferences as a basis for resolving coherence gaps [7]. It has been well established that explanatory inferences are generated at coherence gaps when reading text [61,62], and there are a growing number of studies showing they are generated at coherence gaps for picture stories [2,3,4,5,34,37].

The novel contribution of this experiment was the test of the Computational Effort and Coherence Gap Resolution hypotheses in college students. We found that the relationship between the frequency of explanations and the likelihood of segmenting was negative when Bridging Actions were absent (Figure 3A), consistent with the Coherence Gap Resolution hypothesis. To our knowledge, this was the first study to provide evidence that students segment and shift to generate a new event model when they are unable to generate an explanation to map actions. We also conducted exploratory analyses on other strategies identified using the coding scheme. We found that the relationship between paraphrases and segmentation was positive when Bridging Actions were absent (Figure 3B). The increase in scene paraphrases at event boundaries may have reflected resources devoted to laying the foundation at the start of a new event [63]. We elaborate more on this possibility in the General Discussion.

## 4. General Discussion

### 4.1. Theoretical Implications of This Study

This study examined how viewers coordinate mapping and shifting processes when comprehending picture stories. SPECT assumes that the back-end mechanism of mapping evaluates the coherence of incoming information with the current event model as viewers extract new information on each fixation and each new picture in a picture story [13]. Viewers segment and shift to create a new event model when the incoming information cannot be mapped onto the current event model. However, the precise relations between processes related to mapping and shifting have not been sufficiently empirically explored. We employed the three-pronged method [36] to determine how viewers coordinate the shifting process of event segmentation (Experiment 1) and the mapping process of bridging inference generation (Experiment 2) by manipulating the presence/absence of Bridging Actions to create coherence gaps.

We found that both the likelihood of perceiving an event boundary and the number of explanatory inferences increased when Bridging Actions were absent. The segmentation data replicated prior work showing that people segment at coherence gaps [34]. The results from the analysis of explanations were consistent with prior work showing that people are more likely to mention Bridging Actions when those pictures are absent than when they are present [5].

Importantly, we found that there was a negative relationship between the likelihood of segmenting and the frequency of explanations in the Bridging-Action absent condition, consistent with the Coherence Gap Resolution hypothesis. This suggests that people are more likely to segment and shift to construct a new event model when they cannot successfully map actions with a bridging inference. Unexpectedly, there was a positive relationship between explanations and segmentation likelihood in the Bridging-Action present condition when people did not have to make the bridging inference. This pattern was consistent with the notion of Computational Effort; however, our predictions were specific to the Bridging-Action absent condition. Taken together, the results lent some support for both hypotheses, which was contingent on the presence of a coherence gap.

**Figure 4:**
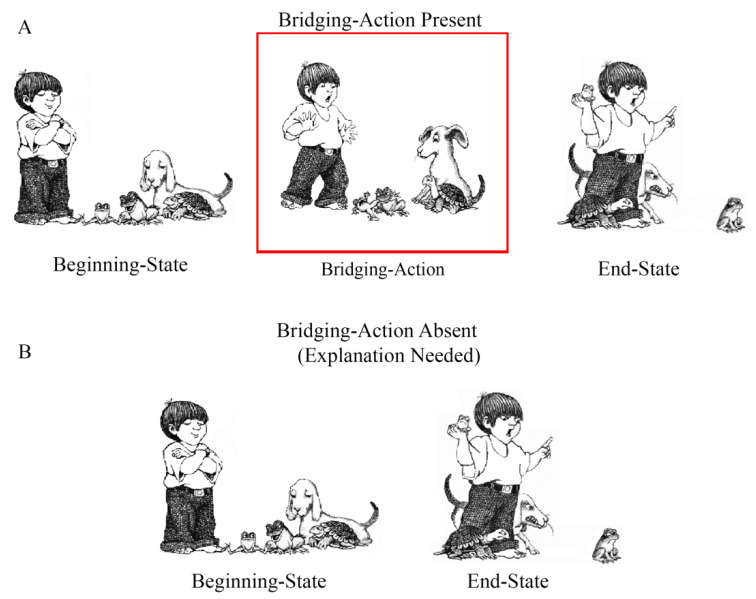
Example episode containing a Beginning State, a Bridging Action, and an End State picture. This target episode came from *One frog too many* [42],which was used in the present study. (**A**) shows the Bridging-Action present condition and (**B**) shows the Bridging-Action absent condition.

What factor(s) can account for the expected and unexpected relationships we observed between think-aloud strategies and segmentation? One possibility is that the degree to which the Beginning and End State pictures were causally related after removing the Bridging Action affected viewers’ ability to generate explanations and the likelihood of segmenting. For instance, Myers et al., (1987) showed that the extent to which readers engage in bridging inference processes depends on the causal distance between events. Two events tend not to be bridged with an inference if they are too causally distant. The materials used in this study were naturalistic picture stories; therefore, we did not control the level of causal relatedness between the Beginning and End State pictures. This episode-specific variability in the degree of causal relatedness between the Beginning and End State pictures when Bridging Action pictures were absent may have affected the likelihood of explaining, paraphrasing, segmenting, and the nature of their relationship. Consider the episode shown in Figure 1. This episode had a high number of explanations and a low likelihood of segmentation. See also Figure 3A. The Beginning State picture shows the frog in the air and the End State shows the saxophone player searching for the frog in the saxophone. The causal distance between these actions is arguably relatively close. There are few actions that can happen between them, which may have facilitated the explanation that the frog jumped into the saxophone. Generating this explanation could have closed the coherence gap and reduced the likelihood that this image was perceived as an event boundary. In contrast, consider the episode in Figure 4, which had a low number of explanations and a high likelihood of being perceived as an event boundary. See also Figure 3A. The Beginning State shows the boy and his pets gathered and the End State picture shows the boy scolding the big frog while the little frog is crying. The actions in the Beginning and End State pictures in this episode are arguably more causally distant than those in Figure 1. In Figure 4, there are relatively more possible actions that could happen between the Beginning and End State pictures, and consequently, there is less support for explanations that connect the two pictures. Shifting may be more likely to occur when there is less support for identifying specific actions that map between the Beginning and End State pictures. While the manipulation of the Bridging Action presence creates a causal coherence break [61], doing so can also create unintended variability in the causal relatedness between the explicitly conveyed events (i.e., the Beginning and End State pictures). It is important to develop approaches to quantify this variability in future research.

Furthermore, the inability to generate explanations to bridge the gap could have increased the need to describe the actions conveyed in the pictures as participants laid the foundation for the subsequent event model, hence the positive trend between the likelihood of segmentation and picture paraphrasing shown in Figure 3B. Loschky et al., (2015) lend some credibility to this possibility. They manipulated the amount of a film clip participants saw before viewing a critical shot. To understand the critical shot in the clip, viewers had to generate an inference to connect it with prior information they watched in the movie. Those who saw more of the clip, and thus had a richer event model of the narrative, were more likely to generate an inference that causally connected the two shots in their think-alouds. Those participants were also less likely to perceive an event boundary on the critical shot and they were less likely to describe the contents of the shot. Conversely, those who watched less of the film clip prior to the critical shot and, thus, those who had a poorer event model of the narrative, were less likely to generate the inference; they were more likely to perceive the critical shot as the start of a new event and they were more likely to describe the contents of the shot. Taken together with our results, it seems that explaining actions goes together with inference generation, as part of mapping information onto the viewer’s current event model, whereas shifting (i.e., segmenting) goes together with describing the contents of the scene as part of laying the foundation for a new event model.

We found a positive relationship between the likelihood of segmenting and the frequency of explanations in the Bridging-Action present condition (Figure 3A). This finding was unexpected because the competing Coherence Gap Resolution and Computational Effort hypotheses focus on the direction of the relationship when Bridging Action pictures are absent. One possibility is that differences in the complexity of the background and foreground actions may help explain the nature of the relationship between explanations and segmentation in the present condition. For example, the foreground in Figure 1 shows the musician searching for the frog in the saxophone. We selected the target episodes in the stories because the foregrounded actions required a bridging inference when the Bridging Action picture was absent. The ease of comprehending the foregrounded actions in the present condition may have led viewers to attend more to the background actions, which influenced the generation of explanations about why those actions were happening (e.g., ‘why are the other musicians angry at the saxophone player?’). The effort required to explain the background actions may have increased the likelihood of shifting to create a new event model, consistent with a modified version of the Computational Effort Hypothesis applied to the present condition. Future research should norm the complexity of the foreground and background actions, replicate this unexpected effect, and examine how foreground and background actions influence explanations and event segmentation.

The further refinement of SPECT regarding the coordination of mapping and shifting requires studies that assess the time course of event segmentation and bridging inferences. Namely, if inference processes precede, delay, or prevent the perception of an event boundary, then it would be consistent with the explanation that viewers segment at coherence gaps when mapping fails. Prior work found that generating bridging inferences in text comprehension takes between 800 and 1000 milliseconds [64]. In contrast, recent work using event related potentials (ERPs) in comics has shown that viewers may generate inferences much earlier (between 400–800 milliseconds after viewing a picture). For instance, the P600 component is larger when viewers generate bridging inferences [2]. Interestingly, ERP data in the discourse comprehension literature also suggest that the P600 may be a marker for event segmentation processes [65]. Thus, it is possible that explanations and segmentation processes operate in parallel until the moment that an individual either connects the information between pictures or segments and shifts to generate a new event model.

The further refinement of SPECT could also come from exploring individual and group-level differences in inference generation and event segmentation. Differences such as in age, working memory capacity, general knowledge, domain-specific knowledge, reading skill, and experience reading picture stories contribute to how viewers engage in inference generation and segmentation processes [66,67,68,69,70,71,72]. For instance, older adults, poor comprehenders, and individuals with lower working memory capacities may be less likely to explain actions and more likely to perceive event boundaries at a coherence gap [67,72]. One important individual difference to consider with respect to visual narratives is exposure to the medium [73]. Visual narratives follow conventions and it has been well documented that individual differences in experience with visual media (both consumption and production) can affect participants’ comprehension of picture stories [2,74,75]. There is a rich history of research exploring individual and group-level differences in the context of reading and we hope to see more research on this issue in the future when people comprehend picture stories. The results of the present study suggest that this research should focus on the relationship between mapping and shifting.

It is also important to consider the extent that culture affects the comprehension of picture stories [74,76]. Cultural differences influence how viewers engage front-end processes of attentional selection and back-end processes involved in comprehension [77,78]. Moreover, there are cultural variations in the conventions of visual narratives that vary across North America, Europe, and Asia [76], which could have important implications on comprehension. As such, replicating this study with samples from different cultures is warranted.

Finally, these results also have important implications for Event Segmentation Theory [19], which describe a different set of mechanisms than those proposed by SPECT to account for event segmentation. Specifically, Event Segmentation Theory says that the event model generates predictions for the near future and that people segment and shift to create a new event model when there are spikes in prediction error. Unlike SPECT, Event Segmentation Theory does not contain an explicit mechanism to support bridging inference generation such as mapping. We found that explanations and segmentation were negatively associated when Bridging Actions were absent, which indicates that backward mapping [35], in addition to prediction error, affects shifting.

### 4.2. Limitations

One limitation of this study was that the think-aloud and segmentation data came from different participants at different institutions. Some of the procedures, such as the viewing distance, differed between institutions. We do not think that such minor variations between labs affected our key results, though it is important to note that differences in viewing distance can be important for the lower-level perception of sensory information such as on visual texture [79].

In addition, while it is common to collect think-aloud and behavioral data from independent samples in the context of the three-pronged method [36], it is also possible to collect these data from the same participants [80]. Future work should examine the relationship between explanations, picture paraphrases, and segmentation on a participant-level basis. That is, the same participants could produce segmentation and think-aloud responses on consecutive viewings of the same picture stories. This would afford a direct assessment of the relationship between explanations, paraphrases, and event segmentation.

### 4.3. Conclusions

SPECT assumes that bridging inference processes support mapping and that segmentation processes support shifting [13]. We examined how people coordinate segmentation and bridging inference processes by manipulating the presence/absence of Bridging Action pictures in picture stories. When experiencing a coherence gap, viewers were more likely to segment pictures that afforded fewer explanations and more paraphrases. These results suggest a complex relationship between paraphrases, explanations, and event segmentation. Specifically, explanations may promote mapping and reduce the likelihood that viewers perceive a new event. In contrast, paraphrases may reflect laying a foundation after segmenting (i.e., shifting to build a new event model). Thus, the present pair of studies represent the first empirical confirmation of SPECT’s critical assumption that the processes of event segmentation and inference generation inform shifting and mapping and the assumption that viewers segment when they fail to map incoming information onto the event model.

## Figures and Tables

**Table 1:** Descriptions of coding categories for the think-aloud responses and example responses for each category.

Strategy	Description	Example
Explanations	Statements that provide reasons why events happened. These could come from prior events or from prior knowledge	*The musicians are unhappy because the frog jumped into the saxophone*
		*He tipped the saxophone upside down because it was plugged*
Predictions	Statements that reflect the anticipation of future events	*The frog is going to cause some trouble*
Associations	Statements about the setting or the character that are not explicitly conveyed in a picture	*This is the woods next to the boy’s house.*
		*The boy must be about 10 years old.*
Picture Paraphrasing		
Narrative Descriptions	Statements that specify the actions that are conveyed in a picture	*He is looking inside the saxophone* *The bandmates are annoyed*
Picture Descriptions	Statements that describe objects and their spatial arrangement in a picture	*The trumpet player has his finger over his mouth* *The drum is below the man*
Metacognitive Statements	Statements that reflect participants’ understanding of the story	*I do not know what is going on*
Evaluations	Statements of whether the participant does or does not like the content of the story	*Very cute story*
Errors	Statements that do not correctly identify the story content. Most of these were misidentifications of characters in the study	*The frog is trying to get into the beehive.*
Other statements	Statements that could not be coded as any of the other categories in the study	*Yeah, I think that’s it for this picture.*

**Table 2:** Mean number of statements for each strategy on End State pictures as a function of the presence/absence of Bridging Actions.

	Bridging Action Presence			
	Absent		Present	
	Mean	SD	Mean	SD
Explanations	1.98	1.63	1.58	1.44
Predictions	0.08	0.35	0.08	0.32
Associations	0.10	0.40	0.11	0.43
Picture Paraphrasing	3.26	2.25	3.33	2.05
Narrative Descriptions	2.47	1.65	2.68	1.67
Picture Descriptions	0.79	1.35	0.65	1.10
Metacognitive Statements	0.15	0.44	0.08	0.31
Evaluations	0.03	0.23	0.03	0.23
Errors	0.02	0.12	0.01	0.11
Other statements	0.17	0.47	0.19	0.51

## Data Availability

We did not preregister this study; however, all the stimuli, data, and analysis scripts in the statistical programming language R are publicly available at https://osf.io/u6bcp/ (Accessed 28 June 2024).

## Supplementary Materials

The following supporting information can be downloaded at: https://www.mdpi.com/article/10.3390/vision8030050/s1.
