# Supplementary material for: Bridging a Gap in Coherence: The Coordination of Comprehension Processes When Viewing Visual Narratives
Source: Vision (Basel). 2024 Aug 30;8(3):50. doi: 10.3390/vision8030050 (PMC11417950; doi:10.3390/vision8030050)
Supplement: Supplementary file 1 [file vision-08-00050-s001.zip › vision-3003207-supplementary.pdf]

Supplementary Materials

Experiment 1

Analysis of Event Segmentation

To determine the random effect structure of the model of event segmentation, we started by fitting the ‘maximal’ model, which included the random intercept of subject, the episode, and the image nested within the episode. We allowed the presence/absence of the Bridging Action to vary for each subject as a random slope effect. We removed the Bridging Action slope as a random effect in the second model, retaining the random intercepts. The first and second model did not differ statistically ($\chi^2(2) = 1.62$, $p = .44$) suggesting that the Bridging Action slope was unnecessary. We removed the random intercept of the episode from the third model so that the third model contained the random intercept of the subject and the image nested within the episode. The second and third models also did not differ ($\chi^2(1) = 0.05$, $p = .83$) which suggests that the by-episode intercept was also not necessary to improve the fit of the model. Thus, we retained the model with the random intercepts of the subject and the image nested within the episode.

Assessing the fit of the logistic model: To assess how well the logistic model of event segmentation behavior was able to classify whether an image was identified as an event boundary on an image-by-image basis and, thus, to provide an estimate of the size of the effect, we fit a receiver operating characteristic (ROC) curve using the model’s estimated values for each End State, which ranged from 0 to 1, and the observed data, which was coded as a 1 if the End State was identified as a boundary and a 0 if it was not. We used the pROC package in R [1]. The area under the curve (AUC) for a model with a perfect ability to discriminate whether an

image was identified as a boundary equal to 1 and a poor model had an AUC equal to 0.5. The analysis of segmentation did well at discriminating images identified as boundaries and non-boundaries; AUC = 0.77 and 95% CI = [0.74 – 0.80]. Thus, segmentation behavior was sensitive to breaks in narrative coherence.

Analysis of Viewing Time

We modeled viewing time on the End State pictures as a function of the Bridging Action presence. We fit three different models with the same random effect structure as in the analysis of event segmentation to determine the random effect structure of the model we retained. We found that the maximal model differed significantly from the model that did not contain the random slope of Bridging Action presence/absence ($\chi^2(2) = 8.92$, $p = .01$). Thus, we retained and reported the results from the maximal model.

Experiment 2

Coding of Think-Aloud protocols

The think aloud statements were parsed into idea units (clauses containing a verb phrase) [2]. The clauses were coded in the context of a larger coding system that involved a variety of different strategies (e.g., picture description, picture narration, explanations predictions, associations, metacognitive statements, questions). These categories were mutually exclusive such that an idea unit could be only coded as one category. Details about the coding scheme are provided in Table S1.

Picture descriptions consisted of statements in which participants described objects and their spatial arrangement in the end-state pictures (e.g., “The boy’s feet are sticking out of the water”, “The frog is on the lily pad”, “The boy is near the hole”). *Narrative descriptions*

constituted statements that specified the events that were conveyed in the stories (e.g., "The boy fell into the pond", "The dog is barking at the beehive"). If the picture clearly depicted a facial expression associated with a basic emotion (e.g., anger, sad, happy, etc.), and a participant stated a basic emotion of a character (e.g., "The frog is mad", "The boy is worried"), it was coded as a narrative description. Both picture and narrative descriptions reflect aspects of paraphrasing; thus, these two codes were combined to create a *picture paraphrasing* variable. *Explanations* specify statements that provided reasons why events happened and could come from explicitly conveyed prior events (e.g., "The boy tripped on the tree root", "The rodent bit the boy's nose") or knowledge-based inferences (e.g., "The boy was too excited when he ran down the hill"). Decisions for each category were made dichotomously (present = 1 vs. absent = 0) and were mutually exclusive such that if a clause was identified as one category (i.e., an explanation), it could not also be identified as another (i.e., a narrative description).

Table S1

Strategies while reading the picture stories.

Strategy	Description of statement	Example Strategy
Explanations	Statements that provide reasons why events happened. These could come from prior events or from prior knowledge	*It bit the boy's nose.* *The boy is holding his nose because the groundhog smells bad.* *The boy got too excited when he ran down the hill.*
Predictions	Statements that reflect the anticipation of future events	*The dog is going to cause some trouble*

		The frog is going to hop away
Associations	Statements about the setting or the character that are not explicitly conveyed in the picture	*This is the woods next to the boy's house.*<br>*The boy must be about 10 years old.*
Picture Paraphrasing		
Narrative Descriptions	Statements that specify the actions that are conveyed in the picture	*He's rubbing his nose*<br>*The boy fell into the pond*
Picture Descriptions	Statements that describe objects and their spatial arrangement in the picture	*The dog is still in the background.*<br>*The frog is on the lilly pad.*
Metacognitive Statements	Statements that reflect participants' understanding of the story	*I do not know what is going on*
Evaluations	Statements of whether the participant does or does not like the content of the story	*Very cute story*<br>*The boy has no right to be mad at the frog.*
Errors	Statements that did not correctly identify the story content in the study. Most of these were misidentifications of characters	*The frog is trying to get into the beehive.*
Other statements	Statements that could not be coded as any of the other categories in the study	*Yeah, I think that's it for this picture.*

Analysis of Explanations

As in Experiment 1, we determined the random effect structure of the model by comparing models with three different random effect structures. The maximal model did not significantly differ from a model that did not contain the by-subject random slope of Bridging Action presence ($\chi^2(2) = 0.65$, $p = .72$), so we removed the effect as a random slope effect. The more parsimonious model did not differ significantly from a model that did not contain the random intercept of the episode ($\chi^2(1) = 0.00$, $p = .99$), so we retained the simpler model that contained the by-subject random intercept and the image nested within the episode.

Analysis of Picture Paraphrases

We modeled the frequency of picture paraphrases the same way we modeled explanations. Again, the maximal model did not differ significantly from a model without the by-subject random slope effect of Bridging Action presence/absence ($\chi^2(2) = 0.38$, $p = .82$) and the more parsimonious model did not differ from a model that only contained the random effect of the subject and the image nested within the episode intercepts ($\chi^2(1) = 0.00$, $p = .99$).

Analysis of Event Segmentation and Think-Aloud Strategies

Analysis of Event Segmentation and Explanations: The model included the fixed effects of Bridging Action presence, the frequency of explanations, and their interaction. We compared three models with the same random effect structure reported in Experiment 1. We started by fitting the maximal model, which contained the random effect of the subject, the episode, and the image nested within each episode. It also contained the interaction between the subject and the Bridging Action presence. It did not differ significantly from a model that did not contain the random slope of Bridging Action presence ($\chi^2(2) = 1.73$, $p = .42$). The second

model also did not differ from a model that did not contain the random intercept of episode ($\chi^2(1) = 0.06$, $p = .81$). Thus, we retained and reported the model that contained the random intercept of the subject and the image nested within the episode.

Exploratory Analysis of Event Segmentation and Picture Paraphrasing: The model included the fixed effects of Bridging Action presence, the frequency of picture paraphrasing, and their interaction. We started by fitting the maximal model. It did not differ significantly from a model that did not contain the random slope of Bridging Action presence ($\chi^2(2) = 1.68$ $p = .43$). The second model also did not differ from a model that did not contain the random intercept of episode ($\chi^2(1) = 0.12$, $p = .73$). Thus, we retained and reported the model that contained the random intercept of the subject and the image nested within the episode.

References

[1] X. Robin *et al.*, “pROC: an open-source package for R and S+ to analyze and compare ROC curves,” *BMC Bioinformatics*, vol. 12, pp. 1–8, 2011.

[2] T. Trabasso and J. P. Magliano, “Conscious understanding during comprehension,” *Discourse Process.*, vol. 21, no. 3, pp. 255–287, 1996.
